# Challenges with sling use following shoulder surgery: the patients' perspective

**DOI:** 10.1016/j.jseint.2025.04.016

**Published:** 2025-05-15

**Authors:** Tom C. Galetti, Mihir M. Sheth, George C. Branche, Cyrus M. Press, Michael V. Narvaez, David J. Novak, Brent B. Wiesel, Sameer H. Nagda

**Affiliations:** a University of California, Berkeley, Berkeley, CA, USA; b The Anderson Orthopaedic Clinic, Alexandria, VA, USA; c Centers for Advanced Orthopaedics, Woodbridge, VA, USA; d OrthoVirginia, Fairfax, VA, USA; e Department of Orthopaedic Surgery, MedStar Georgetown University Hospital, Washington, DC, USA

**Keywords:** Shoulder arthroscopy, Shoulder arthroplasty, Shoulder sling, Recovery, Sleep, ADLs

## Abstract

**Background:**

The purpose of this study was to survey patients who had recently undergone shoulder surgery to assess the challenges they faced while immobilized in a sling and how they overcame those challenges, in order to improve future patient outcomes and satisfaction.

**Methods:**

A retrospective survey, completed 3 months after surgery, asked patients to detail the activities of daily living (ADLs) they found to be challenging while immobilized. Patients across multiple sites quantitatively and qualitatively described their problems and discussed both the amount of help required and the individual methods to complete ADLs. Statistical analysis was performed using an unpaired, two tailed T-test.

**Results:**

Three hundred patients noted the most difficult activities while living with the sling were sleeping, bathing, and dressing. 84.5% of patients had difficulty sleeping for an average of 6.5 weeks. 51.8% of patients needed help bathing, 47.5% of patients required help drying off, and 71.1% of patients required help dressing while immobilized. Before completing ADLs on their own, patients required 10.5, 11.5, and 14.8 days of help to bathe, dry, and dress, respectively. Though not statistically significant, patients required less help completing ADLs if they had surgery on their nondominant arm or prior shoulder surgery.

**Conclusion:**

Sleeping is difficult for most patients while immobilized. Bathing, drying, and dressing require an adaptation period of 2 weeks until patients can complete these activities more independently. Informing patients of challenges they will face while immobilized will help set expectations and may lead to improved clinical outcomes.

Sling immobilization to promote soft-tissue healing is ubiquitous in shoulder surgery, and compliance with immobilization has been shown to improve rotator cuff healing and affect clinical outcomes.^3^^,^^13^ Despite the benefits of sling use to clinical results, they can be a source of discomfort and inconvenience to patients. In some cases, the impact of difficulty with postoperative sling immobilization can influence the timing or even whether to perform an otherwise indicated shoulder surgery.

While there have been a number of studies on sling compliance,^3^^,^^8^ the correlation of healing with sling use,^3^^,^^5^^,^^11^^,^^13^ and the type of sling,^2^^,^^4^ there has been less research into the challenges patients face and the strategies they use to manage performing activities of daily living (ADLs) that are difficult with sling use. Understandably, many common preoperative patient questions surround ADLs and activity restrictions.^6^^,^^9^ Previous patients may be the best source of this information on this topic because of their direct experience. While their experience could be learned during clinical visits, that information may not always reach future patients. A synthesis of previous patients' shared experience could help future patient counseling on expectations for recovery, formulation of effective postoperative instructions, and overall patient satisfaction with their surgery.

The primary objectives of this study were to use open-ended questions to understand the most challenging tasks patients faced while required to wear a sling and strategies they used, including the amount of help needed to manage performing them. The secondary objectives were to learn how preparation materials, hand-dominance, and physical therapist involvement influenced patient experience with sling use. The overarching goal in gathering this information was to improve patient preparedness and satisfaction with the recovery process following their shoulder surgery.

## Materials and methods

### Study design and inclusion/exclusion criteria

An Institutional Review Board for the institutions involved deemed this study not human subjects research, and no further Institutional Review Board approval was required. Patients who underwent primary anatomic total shoulder arthroplasty arthroscopic rotator cuff repair (RCR), or arthroscopic labral repair and required use of a 30° abduction sling for a minimum of 4 weeks were preoperatively notified about the goals of the study and later asked to complete the “Shoulder Surgery Recovery Survey” at a routine, 3-month follow-up visit. The surveys were completed either on paper or online after the visit. Patients undergoing reverse shoulder arthroplasty were not included, as some participating surgeons only required sling use for 2 weeks after reverse shoulder arthroplasty, which was significantly less than other requirements after other operations. Ultimately, there were 371 eligible patients from six surgeons and multiple institutions over a 14-month period from April 2022 to June 2023.

### Shoulder surgery recovery survey

An open-ended questionnaire (Supplementary Appendix S1) was designed to achieve the study objectives. Three main aspects of patients' experience with ADLs while wearing a sling were: which ADLs were most difficult, how long they were difficult, and what strategies they used to manage that difficulty. In addition, patients were asked if and for how long they required help to perform particular activities. Questions 1-4 inquired about surgery type, duration of required sling use, surgeon, and whether the patient lives alone. Questions 5 and 6 were open-ended questions asking the three most difficult ADLs while wearing the sling, and how patients managed these tasks. Questions 7-22 focused on specific data points related to duration and coping strategies for difficulty sleeping, bathing, dressing, and cooking. The remainder of questions were focused on the secondary study objectives, including items related to preparedness, hand dominance, prior shoulder surgery, and assistance of a physical therapist.

### Data analysis

Qualitative data, such as the strategies used to cope with difficulty performing ADLs, were first placed into discrete categories. Descriptive statistics were used to report the frequency of answers falling into a previous category and other quantitative data gathered from the study. A Student's t-test and a chi-square test were used to evaluate the influence of demographic and surgical factors on duration and severity of difficulty with ADLs for continuous and categorical data, respectively. For this study, alpha was defined as <0.05.

## Results

### Study cohort

Three hundred of 371 (80.8%) eligible patients completed the survey; the 71 patients who did not either declined to participate or never filled out the survey despite initially agreeing to. The vast majority of patients had undergone arthroscopic RCR (248; 82.7%). The mean age of patients was 58.0 ± 12.6 years. There were 146 (48.7%) males and 139 (46.3%) females; 15 patients did not specify their gender (Table I). The mean duration of sling use was 5.6 ± 2.2 weeks, and on a scale of 1-10, with 10 indicating the greatest difficulty, patients rated their time with the sling 5.8 out of 10. There were 50 (16.6%) patients who lived alone, of which 35 (70%) had someone stay with them for a mean 16.6 ± 28.9 days. There were 225 (75%) patients who had no prior shoulder surgery.

**Table I:** Study participant demographics.

Demographic breakdown	Number of patients (% of all respondents)	Average age ± SD (yr)
Total respondents	300 (100)	58.0 ± 12.6
Male∗	146 (48.6)	58.0 ± 11.8
Female∗	139 (46.3)	58.0 ± 13.3
Arthroscopic RCR	248 (82.7)	59.0 ± 12.8
Male	117 (39.0)	58.9 ± 13.5
Female	118 (39.3)	59.0 ± 12.1
Anatomic TSA	31 (10.3)	65.8 ± 11.0
Male	16 (5.3)	67.9 ± 11.1
Female	14 (4.7)	63.4 ± 11.1
Arthroscopic labral repair	21 (7.0)	34.5 ± 12.6
Male	13 (4.3)	37.1 ± 14.5
Female	7 (2.3)	29.7 ± 10.5

*SD*, standard deviation; *RCR*, rotator cuff repair; *TSA*, total shoulder arthroplasy.

Patient demographics viewed by surgery and gender.

∗ 15 respondents chose not to disclose their gender, so gender breakdowns do not sum to the total. By surgery, 13 arthroscopic RCR, patients, 1 anatomic TSA, 1 arthroscopic labral repair patient chose not to disclose gender.

### Frequency and duration of difficulty with activities of daily living

In response to a free-response question asking the three most difficult aspects of daily life, the most frequent responses were sleeping (178; 59.3% of all patients), dressing (172; 57.3%), bathing (106; 35.3%), cooking (51; 17.0%), and driving (51; 17.0%). There were multiple difficult ADLs that emerged in the free-response answers that were not specified in the later multiple-choice questions, including: combing hair (32; 10.7%), toileting (31; 10.3%), eating (30; 10.0%), typing (22; 7.3%), and cleaning (13; 4.3%). In both open-ended and closed-ended questions, sleeping was most frequently rated as the single most difficult ADL (34.0% and 46.8% of patients, respectively). In a close-ended ranking of ADLs, following sleeping, the remaining ADLs were listed from hardest to easiest: bathing, dressing, cleaning, cooking, driving, and grocery shopping.

Questions 7-9 were targeted at specific characteristics of difficulty sleeping. For those who had difficulty sleeping, the mean duration of difficulty sleeping was 6.5 ± 3.9 weeks, with 51.5% of patients reporting difficulty for the entire duration of sling use and 33.0% reporting difficulty for some of the duration of sling use. For patients who reported some difficulty sleeping, but for less than the entirety of sling use, the mean duration of difficulty was 4.0 ± 2.8 weeks. When asked why the sling disturbed sleep, the most frequent responses were difficulty finding a comfortable position (86.7%), the bulkiness of the sling itself (73.8%), and shoulder pain (62.5%).

**Figure 1:**
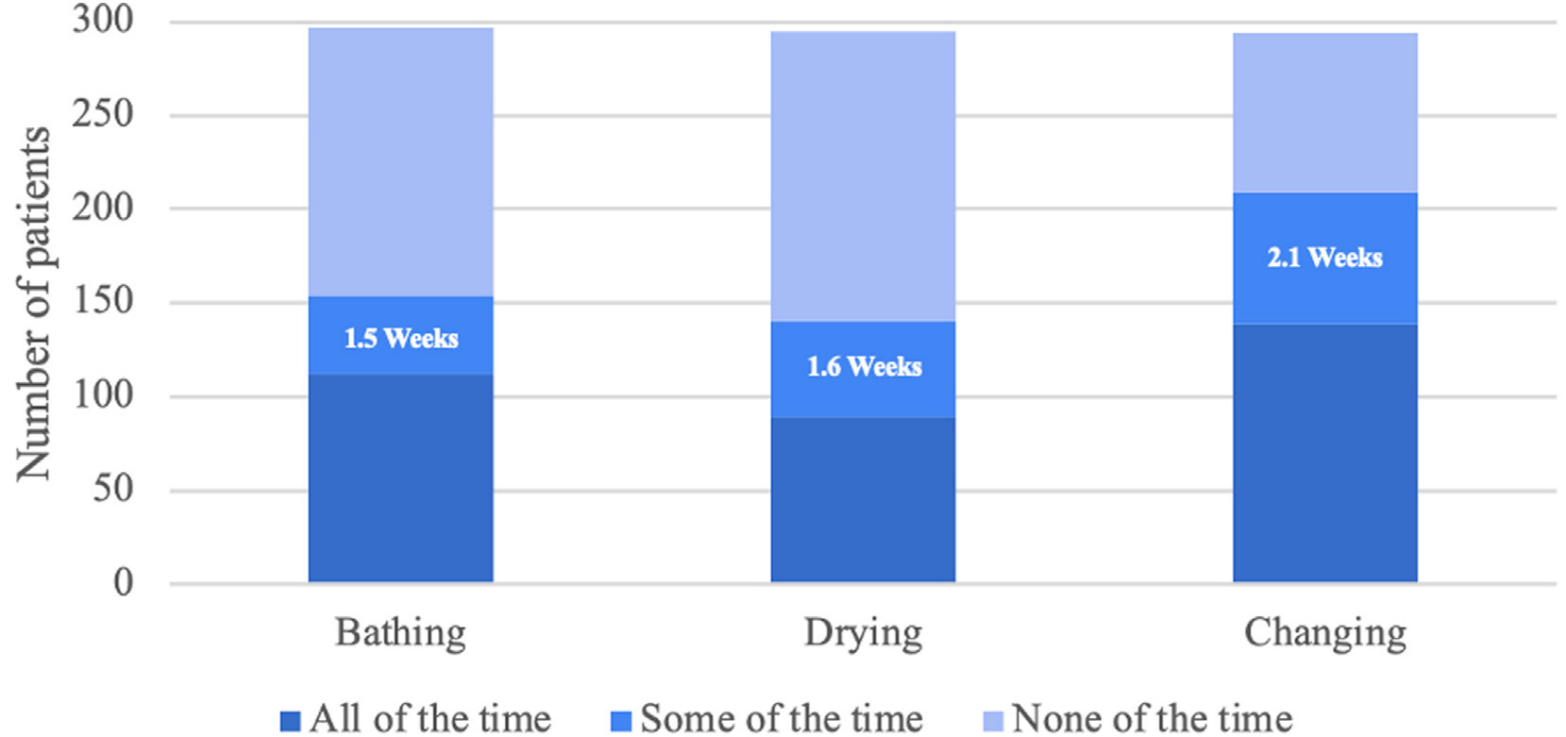
Duration of requiring help with bathing, drying, and changing while using a sling. The mean duration of requiring help from another person for patients who responded “some of the time” is listed within the bar corresponding to that response.

Questions 11-22 were targeted at the frequency and duration of difficulty bathing, drying, dressing, and cooking. A number of patients required at least some help, as 153 (51.5%) patients requested help bathing, 140 (47.5%) patients requested help drying off, and 210 (71.2%) patients requested help dressing. Patients who required help for less than the entire time in the sling required 10.5, 11.5, and 14.8 days of help to bathe, dry, and dress, respectively (Fig. 1). With regard to cooking, 163 (55.8%) tried to cook themselves, but many of these patients required help from another person for some of the preparation (105, 65.2%) and/or received food from outside sources (ie, take-out) (67, 41.6%).

### Strategies to manage difficulty with particular activities of daily living

The open-ended questions 10, 11, 12, 14, 16, and 20 inquired about strategies to help manage difficulty with sleeping, bathing, drying, dressing, and cooking. Generally, patients reported requiring more time to complete ADLs, and further strategies that were reported to be helpful by at least 5 separate patients are listed in Table II.

**Table II:** Strategies to manage difficulty with particular ADLs.

Sleep	Bathing	Drying	Changing clothes
• ***Semi-recumbent or upright positioning (couch, recliner)*** • ***Additional pillows for support*** • ***Pain control with medication or ice*** • **Loosening sling** • Sleeping on the other side	• **Long-handle brush** • **Detachable showerhead** • **Shower chair/seat** • **Purchasing a mesh shower sling** • **Pump-dispenser soap and shampoo** • **Go slowly and take more time**	• ***Go slowly and take more time*** • **Air dry back and surgical area** • **Use the wall to dry the back** • Beach towel • Blow-dryer	• ***Larger size clothing*** • ***Go slowly and take more time*** • **Injured arm into shirt first** • **Elastic waistband pants** • Dressing while seated • Slip on shoes

*ADLs*, activities of daily living.

Italics and boldface denotes response by >50 patients. Boldface denotes response by >10 patients.

The majority of listed strategies for sleeping fell into two categories: semi-recumbent positioning and pain control. Semi-recumbent positioning was achieved through extra or wedge pillows (64.9%), recliner chairs (22.0%), and sleeping on a couch (2.7%). Pain control was achieved through pain or sleep medication (32.1%) and ice (8.4%). There were also patients who reported relief with loosening the sling (4.0%) and sleeping on the good side (2.7%).

For bathing, patients reported multiple strategies that involve purchasing or acquiring helpful items, including a long-handled brush, shower chair, mesh (waterproof) sling, and pump-dispenser toiletries. There were also patients who wrote that they had help predispensing their toiletries before entering the shower. To dry off, there were a variety of responses that did not clearly fall into broad categories; the most common responses were to allow the arm and back to air dry on their own (16.9%) and use a wall to dry the back (7.4%).

Patients reported a variety of strategies to dress themselves. Larger-size clothing was found to be helpful for 31.1% of patients. For upper body clothing, the most frequent strategies were placing the injured arm into the shirt first, leaning forward to put the shirt on, wearing a button-up shirt, and using a modified shirt (ie, magnet, velcro, sleeveless). For lower-body clothing, the most frequent strategies mentioned were elastic waistband pants and slip-on shoes. With regards to wearing a bra, women reported not wearing a bra or clasping the bra in front, using a sports bra or tank tops, or stepping into the bra as methods of safely putting on a bra.

### Statistical analysis of factors that may impact the amount of help required

One hundred and eighty-eight (83.5%) patients for whom this was their first shoulder surgery felt prepared for difficulties associated with sling use. The vast majority cited the physician's written postoperative instructions (78.1%) and preoperative counseling with the care team (71.1%) as their primary resource for preparation; few found online resources to be helpful, with 39 patients (20.9%) listing YouTube as a resource. Among patients who had prior experience with sling use, 87.7% felt better prepared the second time. Those who had prior experience with sling use reported slightly less help with bathing (mean difference, 1.0 days) and dressing (mean difference, 1.8 days) when compared to first-time shoulder surgery patients, though neither difference was statistically significant (*P* = .63 for bathing; *P* = .49 for dressing).

In the overall cohort of 300 patients, 240 (80.0%) felt that sling use on their nondominant arm made it easier to complete ADLs. Patients did, on average, require less help when surgery was performed on their nondominant arm, but the differences in mean duration (<3.5 days) of help required with sleeping, bathing, drying, and dressing did not reach statistical significance, with *P* values of .14, .36, .25, and .32, respectively.

The guidance of a physical therapist was associated with a slightly lower duration of help required with bathing (mean difference 1.3 days), drying (mean difference 0.7 days), and dressing (mean difference 0.1 days) that did not reach statistical or clinical significance (*P* = .38 for bathing; *P* = .68 for drying; *P* = .97 for dressing).

## Discussion

While the findings of this study may be intuitive to experienced shoulder surgeons, the relative severity and duration of difficulty can help identify points of emphasis in preoperative counseling and formulation of patient instructions. Sleep was difficult for most patients, and patients required someone else's help with bathing, drying, and dressing for approximately 2 weeks. These difficulties should be communicated to patients and those who live with them, as they may require additional time or assistance from another person each day to complete these tasks.

Sleep was difficult for 84.5% of patients and was most frequently rated the most difficult ADL; the use of semi-recumbent positioning and pain control measures helped patients sleep. Again, patients needed help bathing and dressing for 10.5 and 14.8 days, respectively. Both were made easier with the use of items that are easy to acquire in advance, including long-handle shower brushes, pump-dispensing toiletries, and larger clothing. Some patients required help drying off for an average of 11.5 days, and others used household items in order to dry themselves without additional help.

The vast majority of patients utilized written physician's instructions or information from preoperative visits to guide them. Physical therapist assistance was not found to change the duration or degree of difficulty with sling use. The overall difficulty of using a sling was greater for dominant-sided and first-time shoulder surgery patients. This can be useful preoperatively for patients requiring bilateral shoulder surgeries; operating first on the dominant shoulder may pose the greatest difficulties for the patient.

Managing patient expectations of the recovery process and maintaining sling compliance are important to patient satisfaction and clinical outcome.^3^^,^^8^^,^^12^ The American Society of Shoulder and Elbow Therapists advocates for the use of caution with regard to upper extremity ADLs during the first 6 weeks postoperatively, as this period is vital to the recovery process.^10^ Patients' ability to anticipate details of the postoperative course can help them mentally prepare for difficulties, and possibly make arrangements for the number of easily acquired items that could be helpful to them. The benefit of being able to anticipate difficulties is somewhat supported by the finding that 87.7% of patients using a sling for the second time felt better prepared. We hope to use the information in this study to help first-time sling users have the knowledge and materials to minimize discomfort and inconvenience in the postoperative period.

In the authors' review of the literature, there are few previous studies reporting the duration of difficulty with specific activities and strategies patients found effective to help manage them. Dolan et al found that 89% of patients who had an arthroscopic RCR had some degree of sleep disturbance, and 77% resolved by 6 months after surgery.^1^ Livesey et al found that education on the importance of sling use improved sling usage.^8^ Kolade et al provided a survey related to ADLs and designed for discrete answers to 154 arthroplasty patients; they found that patients can expect the ability to perform “waist-level” ADLs by 3 months from surgery, but this was not specific to sling use.^7^

There are multiple limitations to this study. While the survey asked patients what they did to manage particular activities, the answers do not help understand the efficacy and relative efficacy of each strategy. There was heterogeneity in procedure type, age groups, and living situation of the included patients that could have affected the difficulty with sling use. This heterogeneity adds generalizability to the study but may have introduced unrecognized confounding variables. There may have been a recall bias in answering the questions; we attempted to minimize this by informing patients about the survey preoperatively and providing the surveys to them at a standardized time point. The length of the survey may have introduced fatigue that affected answers to later questions and open-ended responses. The design of the survey may also have led patients to include difficulties that were not the direct consequence of sling use and therefore outside the desired scope of the study, such as analgesic regimen. Sling compliance was not included in the study, and ideally, we could have only included patients compliant during their intended period of sling use.

The strengths of this study include the large sample size, high (>80%) response rate, and involvement of multiple treating surgeons, which add to its generalizability. In addition, the qualitative nature of the responses allowed detection of difficulties and strategies that may be perceived as too inconsequential to be discussed in clinical visits but may help other patients.

The future directions of study may include prospectively identifying which strategies are most efficacious and cost-effective, whether or not difficulties vary by surgical procedure, and in which patient demographics added measures to help manage sling use could be helpful. In addition, we hope this study gives provider teams important information to develop patient education materials for future shoulder surgery patients.

## Conclusion

This study was conducted to gain better insight into the common difficulties that patients face while living with a sling after shoulder surgery. Notably, patients undergoing shoulder surgeries that require postoperative sling immobilization have found bathing, drying, and dressing to be difficult while immobilized, and patients required help for approximately 2 weeks before being able to complete these tasks on their own. While immobilized, patients may require additional time and assistance to complete these tasks. Further, the majority of patients found sleeping while immobilized to be difficult. Patients undergoing first-time, dominant-sided surgery may warrant added attention and counseling. Written instructions from the physician are the most commonly utilized resource for education on sling use strategies, and information on how to safely complete ADLs may increase patient satisfaction and improve clinical outcomes.

## Disclaimers:

Funding: No funding was disclosed by the authors.

Conflicts of interest: None. Dr. Wiesel report spublishing royalties, financial or material support from Wolters Kluwer Health-Lippincott Williams & Wilkins outside of the submitted work. Dr. Nagda reports IP Royalties from Aevumed as well as being a paid presenter or speaker and research support from Stryker outside of the submitted work. The other authors, their immediate families, and any research foundation with which they are affiliated have not received any financial payments or other benefits from any commercial entity related to the subject of this article.

## References

[1] Dolan M.T., Lowenstein N.A., Collins J.E., Matzkin E.G. (2022). Majority of patients find sleep patterns return to normal 6 months following rotator cuff repair. J Shoulder Elbow Surg.

[2] Gao J.-H., Zhou J.-Y., Li H., Li H.-Y. (2023). Sling versus abduction brace shoulder immobilization after arthroscopic rotator cuff repair: a systematic review and meta-analysis. Orthop J Sports Med.

[3] Grubhofer F., Ernstbrunner L., Gerber C., Hochreiter B., Schwihla I., Wieser K. (2022). Effect of abduction brace wearing compliance on the results of arthroscopic rotator cuff repair. JB JS Open Access.

[4] Hollman F., Wolterbeek N., Zijl J.A.C., Van Egeraat S.P.M., Wessel R.N. (2017). Abduction brace versus antirotation sling after arthroscopic cuff repair: the effects on pain and function. Arthroscopy.

[5] Keener J.D., Galatz L.M., Stobbs-Cucchi G., Patton R., Yamaguchi K. (2014). Rehabilitation following arthroscopic rotator cuff repair: a prospective randomized trial of immobilization compared with early motion. J Bone Joint Surg Am.

[6] Khalil L.S., Castle J.P., Akioyamen N.O., Corsi M.P., Cominos N.D., Dubé M. (2023). What are patients asking and reading online? An analysis of online patient searches for rotator cuff repair. J Shoulder Elbow Surg.

[7] Kolade O., Ghosh N., Buchalter D., Rosenthal Y., Zuckerman J.D., Virk M.S. (2023). Patterns of limitations in activities of daily living, sleep, and pain in the early postoperative period following total shoulder arthroplasty: a prospective study. JSES Int.

[8] Livesey M.G., Weir T.B., Addona J.L., Curto R.A., Apte A., Hughes M. (2023). The effect of patients’ understanding of sling necessity and home assistance on sling wear after shoulder surgery. Am J Sports Med.

[9] Sudah S.Y., Pagani N.R., Nasra M.H., Moverman M.A., Puzzitiello R.N., Guss M.S. (2022). What patients want to know about shoulder arthroplasty: a Google search analysis. Semin Arthroplasty.

[10] Thigpen C.A., Shaffer M.A., Gaunt B.W., Leggin B.G., Williams G.R., Wilcox R.B. (2016). The American Society of Shoulder and Elbow Therapists' consensus statement on rehabilitation following arthroscopic rotator cuff repair. J Shoulder Elbow Surg.

[11] Tirefort J., Schwitzguebel A.J., Collin P., Nowak A., Plomb-Holmes C., Lädermann A. (2019). Postoperative mobilization after superior rotator cuff repair: sling versus no sling: a randomized prospective study. J Bone Joint Surg Am.

[12] Waljee J., McGlinn E.P., Sears E.D., Chung K.C. (2014). Patient expectations and patient-reported outcomes in surgery: a systematic review. Surgery.

[13] Weir T.B., Enobun B., Livesey M.G., Sood A., Apte A., Hughes M. (2023). Increased actual sling wear is associated with better early patient-reported and image-based outcomes after shoulder surgery. J Shoulder Elbow Surg.

