# Supplementary material for: Challenges with sling use following shoulder surgery: the patients' perspective
Source: JSES Int. 2025 May 15;9(4):1385–9. doi: 10.1016/j.jseint.2025.04.016 (PMC12434999; doi:10.1016/j.jseint.2025.04.016)
Supplement: Supplementary Appendix S1 [file mmc1.docx]

Post Shoulder Surgery Questionnaire

1. **What procedure did you have done? Circle one option below.**

Total anatomic shoulder replacement / Rotator cuff repair / Repaired labral tear

1. **For how long were you required to wear the sling? Circle one option below.**
   1. 1 / 2 / 3 / 4 / 5 / 6 week(s)
   2. Unsure/can’t remember
   3. Longer than 6 weeks ___________ (specify)
2. **Who was your surgeon?** _____________________________
3. **Do you live alone?** **Circle an option.** Yes/No

If you chose “Yes” for question 4 above, answer question “a” below.

- 1. **Did you have someone come stay with you? Fill out an option below:**

Yes. They stayed for ______ (days) No

1. **Which three aspects of daily life did you find to be the hardest to do while required to wear the sling?**
   1. ____________________________________________________________
   2. ____________________________________________________________
   3. ____________________________________________________________
2. **How did you manage to do the aforementioned tasks?**
   1. ____________________________________________________________
   2. ____________________________________________________________
   3. ____________________________________________________________
3. **Did you have problems sleeping?** **Select an option below** YES/NO

**If you answered “YES” to question 7, answer questions 8, 9, and 10 below**

**If you answered “NO” to question 7, answer question 10 below**

1. **What were the causes of your difficulty sleeping? Select any (or all) that apply:**
   1. The sling itself (positioning, general discomfort of a sling, etc)
   2. Shoulder pain
   3. Effects of pain medications
   4. Restrictions moving your arm
   5. Something else (comment below) ___________________________________________
2. **How long did your difficulty sleeping last for?** _____________ (weeks)
3. **What did you find most helpful in allowing you to sleep? (Positioning/pillows, medication, etc)**

__________________________________________________________________

1. **Did you need assistance bathing?** **Circle one of the three options below.**

No Yes, for the entire time in the sling Yes, for _______ (days)

1. **If you answered “No” or “Yes, for _____ (days)”: How did you manage to bathe yourself without moving your arm?** ___________________________________________________
2. **Did you need assistance drying yourself off? Circle one of three options below.**

No Yes, for the entire time in the sling Yes, for _______ (days)

1. **If you answered “No” or “Yes, for _____ (days)”: How did you dry yourself off with just one arm?** ________________________________________________________________
2. **Did you need assistance getting dressed?** **Circle one option below.**

No Yes, for the entire time in the sling Yes, for _______ (days)

**If you answered “No” or “Yes, for _____ (days)” answer questions 14, 15, 16, and 17 below**

**If you answered “Yes, for the entire time in the sling,” skip to question 18 about cooking**

1. **In general, how did you get dressed without any help?** __________________________________________________________________
2. **Were there any noticeable differences between garments (like a pullover sweater vs a zip up jacket, or putting on a t shirt vs pants)?** Yes/No

If you selected Yes, please explain below. ______________________________________________

1. **How did you work around the sling when putting on a bra? Circle one option below**

I didn’t have to do this / I did this by… (explain below) ________________________________________________________________________

1. **Did you purchase any special garments or make any modifications to your clothes in order to make changing easier?** **Circle one option below.** No / Yes. Specifically, I bought/altered… (describe below) ________________________________________________________________________
2. **Did you try to cook for yourself while in the sling?** **Circle one.** Yes/No

**If you answered “Yes,” please answer questions 19, 20, 21, and 22**

**If you answered “No,” please answer questions 21 and 22**

1. **What were you NOT able to do in the kitchen (ex: cooking, cutting/chopping, opening jars, reaching for plates, washing dishes, etc)?** __________________________________________________________________
2. **For the things you COULD do, how did you adapt to doing them with just one arm?** __________________________________________________________________
3. **Did you have someone cook at home for you? Circle one:** Yes No
4. **Did you have food delivered to you by outside sources (food delivery programs, family, neighbors, friends)? Circle one:** Yes No
5. **In general, how bothersome was the use of the sling? Try to be holistic and objective when answering. If you got used to it over time (or perhaps you developed a rash from the material and it got worse with time), include that in the comments section below.**
   1. Scale 1 (I hardly noticed it) to 10 (it was the worst thing in the world; I wanted to burn it). Score ________
   2. General comments: ____________________________________________________________
6. **Please rank the following seven activities from hardest (1) to easiest to do (7) with a shoulder sling. Place the rankings on the lines.**

___ Sleeping ___ Bathing ___ Changing ___ Cooking ___ Cleaning ___ Grocery Shopping ___ Driving

1. **Did you see a physical therapist while in the sling? Circle one below.** Yes/No

**If you answered “YES,” please answer question 26**

**If you answered “NO,” please skip question 26**

1. **Did the physical therapist help you learn how to perform any daily tasks at home? Select one:** Yes No

**Please answer 27 or 28, depending on your handedness.**

1. **If this was your DOMINANT arm: Do you think performing these daily tasks would have been easier if the surgery had been on your non dominant arm? Select one:** Yes No
2. **If this was your NON DOMINANT arm: Do you think performing daily tasks would have been harder if the surgery had been on your dominant arm? Select one**: Yes/No
3. **Is this your first shoulder repair/replacement? Select one:** Yes/No

**If you answered “YES,” please answer questions 30 and 31**

**If you answered “NO,” please go to question 33**

1. **If you were to have the same procedure done on the other side, what would you do differently?** __________________________________________________________________
2. **Do you feel you were adequately prepared before the operation for your time in the sling/the recovery process after the operation?** **Select one:** Yes/No

**If you answered “YES,” please answer question 32**

**If you answered “NO,” please skip to question 35**

1. **If Yes to Question 31: What helped you feel prepared (circle all that apply):**

Doctor’s instructions / Visits with members of your care team (PA, nurse, anesthesiologist, etc) / YouTube videos / Facebook posts / Articles online / Past patients (who shared their experiences with you) / Practicing using or living with the sling before surgery / Other (comment what) ______________________________________

1. **If you answered “No” to Question 29: What, if anything, did you do differently between your first shoulder repair/replacement, and this one, your second? Select one:** I didn’t do anything differently / I did something differently. Specifically, I … (comment below) ________________________________
2. **If you answered “No” to Question 29: Did you feel better prepared this time than you did the first time having already gone through the recovery process once?** **Circle one of the following:** Yes No
3. **Did you find any resources online (YouTube videos, Facebook posts, articles, etc) to be particularly useful in navigating life with the sling? Select one of the following:**

No / Yes, I found… ____________________________________________

1. **Contact information (optional) for any follow up questions:**
   1. Name ______________________
   2. Email address ______________________
   3. Phone number _____________________
2. **Any final questions, comments, or concerns?**
